# An automated method for left ventricular localization and identification of end-systolic and end-diastolic images from cine cardiac MRI

**DOI:** 10.1186/1532-429X-11-S1-P222

**Published:** 2009-01-28

**Authors:** Sotirios A Tsaftaris, Xiangzhi Zhou, Richard Tang, Rachel Klein, Rohan Dharmakumar

**Affiliations:** 1 Department of Electrical Engineering and Computer Science, Northwestern University, Evanston, IL USA; 2 Department of Radiology, Northwestern University, Chicago, IL USA; 3 Northwestern University, Chicago, IL USA

**Keywords:** Normalize Cross Correlation, Propose Identification Method, Unique Global Maximum, Scout Scan, Diagnostic Metrics

## Introduction

A critical component in computing quantitative diagnostic metrics, such as ejection fraction, as well as image segmentation and registration is the accurate identification of the end-systolic (ES) and end-diastolic (ED) frames in cine MRI. Localization of the LV is also important, to assist further analysis (ie., myocardial segmentation). Currently, these tasks are performed in a manual, semi- or fully-automated fashion. Fully-automated methods are desirable since they can eliminate manual labor and inter- and intra-observer variability. Most methods rely on measuring the area of the blood pool in the LV chamber, but they are computationally intensive, susceptible to noise, and require prior localization and segmentation of the LV. An image-driven statistical method is presented that utilizes cross-correlation to detect ES and ED from cine MRI acquired from canines under control conditions.

## Purpose

To develop a fully automated, computationally efficient, post-processing method for reliable LV localization and identification of ES and ED frames from cine cardiac MR images.

## Methods

### Experimental setup and imaging

Short-axis cine cardiac MR images were acquired on Siemens 1.5 T scanner from three canines that were sedated and mechanically ventilated. ECG-gated and breath-held SSFP acquisitions were prescribed over the mid-ventricle following scout scans at various temporal resolutions (TRes:7–153 ms). Scan parameters: voxel size = 1.2 × 1.2 × 6 mm^3^; flip angle = 60°; TR/TE = 3.5/1.8 ms.

### Image processing

Each cine set was denoted as I(x, y, t), where x, y are pixel locations (NxM), and t denotes 1,..., F phases. Each image was smoothed by convolution with a Gaussian filter (width = 10 and sigma = 1). The normalized cross correlation is computed between all images, giving an FxF matrix C(i, j), (i, j corresponds to image pairs). The i, j corresponding to the minimum of C are the ES, ED images, since they are uncorrelated in the heart region (high motion). Using the same principle, the LV chamber can be localized by finding the temporal variation of I(x, y, t), as matrix V (NxM). V was then denoised using a Gaussian and binarized (Otsu's method). The centroid of the largest connected component was chosen as the LV center. The proposed identification method was compared against LV blood volume segmentation methods. A seeded-region-growing method (SRGM) was chosen for segmentation instead of level-set-active-contours since it proved more robust to noise and the limited spatial resolution of the cine MRI images.

### Data analysis

Cine images with varying TRes were tested and additive-white-Gaussian-noise was introduced to demonstrate robustness. The seed location for SRGM was manually set inside the ventricle for each image. Average number of frames away from the true ES, ED images (manually chosen) was used as the performance metric. For each noise level, 40 trials were performed and the results were averaged.

## Results

**Figure 1:**
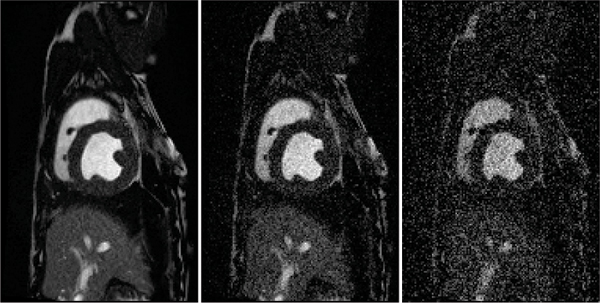
**An image with various levels of noise: left no noise; middle and right additive zero mean noise of 40 and 100 standard deviation, respectively**.

**Figure 2:**
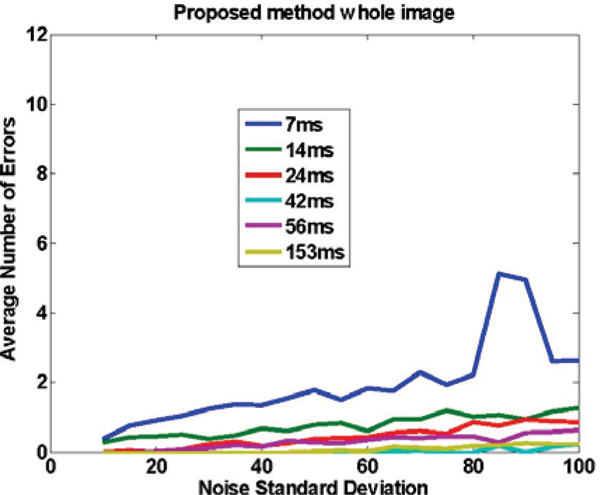
**Accuarcy of the proposed method, as measured by the average number of errors, for different noise levels and for a number of different TRes, when the whole image is used**.

**Figure 3:**
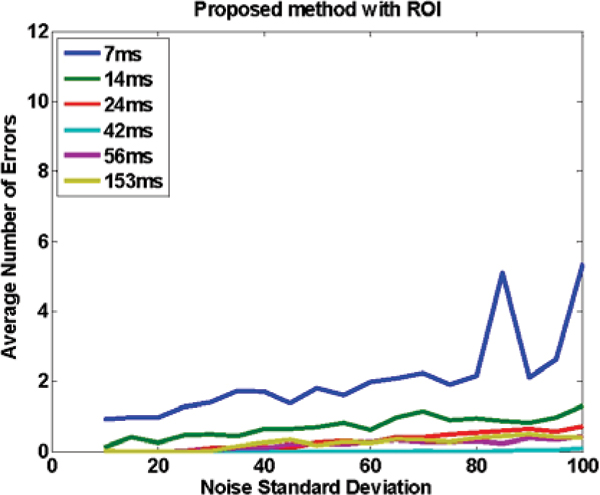
**Accuracy of the proposed method with an ROI that encompasses the heart, as measured by the average number of errors, for different noise levels and for a number of different TRes**. Notice that the performance is similar to Figure 2, illustrating that there is no gain in selecting an ROI, thus demonstrating that the proposed method can be used in the whole image, without needing an ROI selection.

**Figure 4:**
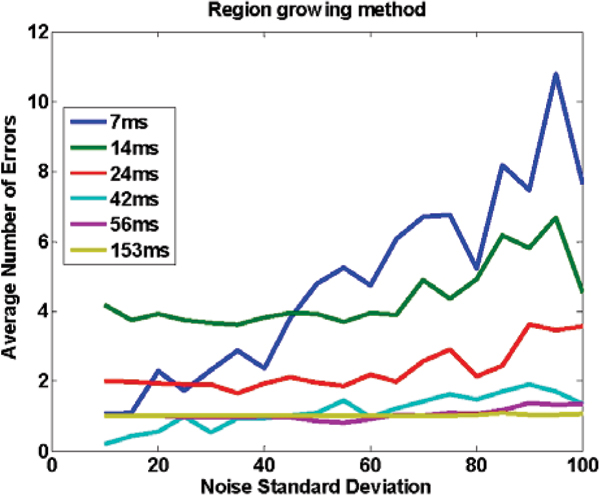
**Accuracy of the seeded region growing method (SRGM), as measured by the average number of errors, for the different noise levels and for a number of different TRes**.

**Figure 5:**
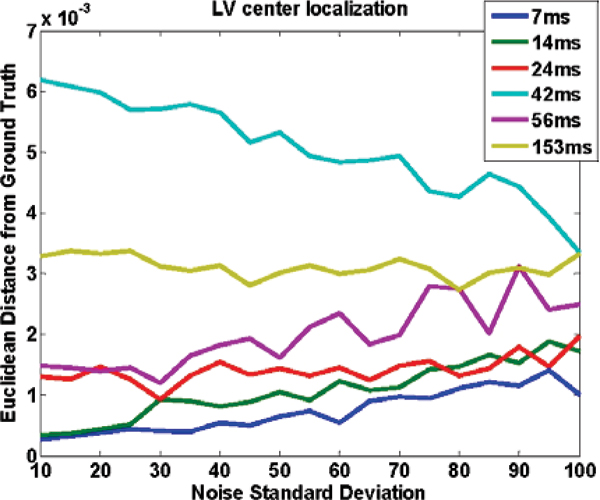
**Accuracy of the proposed method in finding the center of the LV tested for different noise levels, and TRes**. The accuracy is measured as the Euclidean distance from the ground truth provided by an observer. Notice that the y-axis is scaled at 10^-3^ increments.

Figure 1 shows an image with added noise. Figures 2 to 4 demonstrate the performance of the proposed method when the whole image is used (Figure 2), and when a ROI around the heart is manually chosen (Figure 3) and compares it to SRGM (Figure 4). Figure 5 shows the Euclidean distance between the centers found using the proposed and the reader delineation of all cine images.

## Discussion

Pre-selection of an ROI, necessary with other methods, does not offer any significant gain. SRGM underperformed in cases when image noise or TRes was high (14 ms and 24 ms), did not always yield a unique global maximum and minimum, and was not robust against the appearance of papillary muscles, thus hindering the identification of ES and ED. The proposed method reliably identifies the cardiac phases, localizes the LV, without any parameterization (SRGM requires the localization of the seed and the determination of the growing threshold), is computationally elegant (~10 times faster than SRGM), could be easily extended to 4D MRI, and implemented in cardiac image analysis software.

